# The Effect of Intense Flashes of Electric Light upon the Eye

**Published:** 1898-02

**Authors:** Dunbar Roy

**Affiliations:** Atlanta, Ga.; Professor of Ophthalmology in Southern Medical College


### THE EFFECT OF INTENSE FLASHES OF ELECTRIC LIGHT UPON THE EYE.

BY DUNBAR ROY, A.B., M.D., ATLANTA, GA.
Professor of Ophthalmology in Southern Medical College.

While the cases here reported occurred in my practice more than a year ago, they were recently recalled to my mind by reading Professor Haab's article in the July number of *Klin. Monatsblt. für Augenheilkunde,* entitled "Traumatic Macular Changes Produced by Electric Currents." There have been, perhaps, more cases observed than have been reported, of temporary injury to the eye through intense electric flashes, but the rarity of such reports warrants the present presentation, which will at all events call attention to some of the lurking dangers of the electric light.

CASE 1.—Mr. E., aged twenty-nine years, one of the chief superintendents for the city electric light company, consulted me in my office at 1 P.M., May 20, 1895. The history given was that about one hour previous, while trying to adjust some wires, two of them became crossed a short distance in front of his eyes with the result of a blinding flash of light. For a few minutes his vision disappeared, but gradually returned, leaving only a marked burning irritation in his eyes, the reason for the present consultation.

On examining both eyes, a marked pink zone of injected blood-vessels could be seen around the cornea. The conjunctiva of the lids was but slightly irritated. Pupils react normally to both light and accommodation. Vision in both eyes normal. He complained of only a smarting sensation about the lids and a slight amount of photophobia. The use of dark glasses, hot fomentations to the eyes and absolute rest for a few days was the only treatment advised. About two o'clock that night a telephone message was received saying that Mr. E. was suffering so much pain with his eyes that he desired me to come out immediately to his house. On arriving I found the eyes a little more congested than on the previous examination. He was then suffering with violent, aching pains in the eyeball. On a close examination of the cornea this was found to look a little hazy, a condition which was not present at the office examination. There was marked photophobia. Several drops of a 2 per cent. solution of cocain were instilled into the eyes, and also a drop of a weak atropine solution. In about fifteen minutes the patient was perfectly easy, and reported next morning at the office that he had remained so all night, and was even now perfectly comfortable. On examination the cornea was clear and all signs of irritation had subsided. His eyes rapidly returned to their normal state. No changes could be seen with the ophthalmoscope, although the symptoms pointed to decided retinal irritation.

CASE 2.—Mr. M., aged eighteen years, also an employee of the Georgia Electric Light Company. The history of this young man was almost identical with Case 1, with the exception that he was not seen until two days after the injury. An identical electric flash took place just in front of his eyes and he was also blinded for a few minutes. On recovering from the shock he bathed his eyes in cold water, and with the exception of a slight irritation he experienced no discomfort. This irritation persisting, the Company sent him to me for examination.

R. E., V.=$^{20}/_{xxx}$. Improved by no glass. L. E., V.=$^{20}/_{xx}$. Decided ciliary injection in the right eye and very slight in the left. Pupils react normally but appear rather contracted. The cornea of the right eye appeared a little hazy. By ophthalmoscopic examination I must acknowledge that nothing very abnormal could be discovered, although the region of the macula in the right eye may have looked a little cloudy. Neither in this case nor in the first was any scotoma or contraction of the visual field to be found. The patient was ordered to wear dark glasses, use hot fomentations to the eyes, and a drop of atropine solution was instilled into both. He had no further trouble and vision in both eyes became normal. The flash of light in both cases came from wires which carry a voltage of five hundred.

A few cases have been reported where intense electric flashes have caused a temporary blindness, and where the pathologic lesion has been a grayish discoloration in the region of the macula lutea. The action of the snow upon the eyes producing the phenomenon known as "snow blindness" is of no infrequent occurrence in the frigid zones, and the pathologic conditions there found are quite similar to those seen in eyes which have been injured by electric flashes.

The subject of pathologic changes in the macula lutea due to traumatic causes was first given prominence in an article by Professor Haab, of Zurich, at the Seventh International Ophthalmological Congress, held at Heidelberg. Since that time Professor Haab and his associates have continued to study the subject and given at intervals further contributions relating to these traumatic changes.

In 1889, E. Meyer, of Zurich, delivered an inaugural address upon this subject, and in 1896, A. Siegfried, of the same city, and out of the same clinic, published an extensive article upon "The Traumatic Changes in the Macula Lutea of the Retina."

Intense light as the cause of retinal and conjunctival changes has been known to careful observers for a long time. Electric light as a cause is of comparatively recent date, and only since it has become so universally popular for domestic use. One has but to note the dazzling effect produced upon the eyes by the bright reflection from the snow, or the same effect produced when we gaze unaided by tinted glasses upon the sun in partial eclipse, to realize how irritating and blinding such lights are to our visual members. "Snow-blindness" has been known to ophthalmologists for years as also the blinding effect of the bright sunlight upon the eyes. A very interesting discussion of this latter subject with an abstract of all reported cases, can be found in an article by Mackaye, published in the *Ophthalmic Review.* He found that after exposure of the eyes to the sunlight the patients notice a dark spot in the field of vision. This on examination is found to be a positive scotoma which lasts for a certain period of time and then passes away. Mackaye found that this also represented color-scotomata extending over a large area. Such patients will often see objects twisted, producing the condition known as metamorphopsia. The ophthalmoscope will sometimes show no changes, while in others a decided hazy condition will be found in and around the macula occasionally with an accompanying pigmentation. Vision as a rule is reduced, and when this is marked a perfect recovery rarely takes place. Pigmentation of the retina caused by the action of sunlight has been adduced by Monoyer as a cause for the condition known as "retinitis pigmentosa."

Deutschmann has shown by experiment that concentration of direct rays of sunlight upon the retina of a rabbit produces a coagulation of the retinal albumen with an accompanying pigmentation. However, such an analogous condition could not be attributed to the sun. While the action of sunlight from the snow produces a decided retinal irritation it manifests its chief symptoms upon the eye by producing a severe conjunctivitis with sometimes a diffuse keratitis and ulcers of the cornea. It rarely leaves any bad results.

The effect of bright electric lights upon the eye has also been investigated and the result noted. Just what degree of brilliancy it takes to produce injurious effects upon the eye has not been definitely settled. It is true that the ordinary incandescent light certainly seems to be the best for illuminating purposes. It is more brilliant, produces less heat and therefore less particles of combustion, is steadier than gas, and is the nearest approach perhaps to the daily sunlight. However, I must say that experience in the use of both the incandescent light and the Welsbach gaslight has influenced me in a preference for the latter. Of course the latter is more troublesome to keep and therefore has its disadvantages, but when subdued by the use of white shades it certainly makes a most excellent light by which to work. Personally I have used this light for years and now prefer no other.

Hartridge, of London, in an article published in the *British Medical Journal*, 1892, on "The effect of Electric Light upon the Eyes," states that no bad results have ever been observed upon the eyes from the ordinary use of the incandescent light. Although since that publication I have seen no other reports to the contrary, personal observation prevents me from agreeing with the author referred to. A case recently under my observation could be attributed to no other cause.

Mr. H., aged twenty-four years, night clerk in a hotel, consulted me last February on account of a slight irritation in his left eye. History was as follows: Physically he was a perfect specimen of manhood. He worked every night as clerk and bookkeeper under the constant glare of the incandescent electric lights and slept during the day. Up to this time he had never had the least trouble with his eyes. Gave absolutely no history of syphilis nor could any signs be found on the closest physical examination. He had never been sick a day in his life, nor had he ever had the slightest touch of rheumatism. Had never felt better than at the present.

On examination, left eye showed contracted pupil, but no more than the right. It did not respond to light. There was only the faintest circumcorneal zone of congestion. Vision normal but photophobia was present. Right eye was normal. On using atropine in the left eye an incipient iritis was readily diagnosed. It was impossible to dilate the pupil *ad maximum*, although there seemed to be no points of adhesion. The iritic muscles appeared perfectly torpid.

The case proved to be one of prolonged treatment, although at no time were the objective or subjective symptoms severe. Nothing was used but atropine solutions, hot fomentations and blisters to the temple. Leeches finally produced a most rapid amelioration of the symptoms. In this case there was no doubt to my mind that the cause of the attack must be attributed to the electric light. He worked for hours every night with an incandescent drop-light just in front and to the left, so that for hours there was a constant glimmer, especially in the left eye. There was never any trouble with the right eye. Since that time he has had no further trouble as he was transferred after recovery to day duty.

In the last few years there have been a number of young ladies under my care for ocular troubles who were boarding in a large school where incandescent lights were used. I have had to stop some of them from school, and in other cases I have substituted a student's lamp in place of the electric lights with the most happy results. My experience has been that these incandescent electric lights are not suited for a light by which to do constant work.

Hewet, in the *British Medical Journal*, 1893, calls attention to a condition of the eye designated as *electric ophthalmia*. The symptoms of this disease are said to be the same as those of snow-blindness, viz., swollen lids and congested conjunctivæ, later followed by a muco-purulent discharge. This condition is usually found among those employed in electric welding operations, less frequently in those who use the arc lights.

Several cases have been reported of blinding or dazzling of the retina through intense flashes of lightning which show much similarity to those caused by electricity.

Professor Haab, of Zurich, was one of the first to call attention to changes in the retina, especially in and around the macular region from traumatic and constitutional causes, in an article read before the Seventh International Ophthalmological Congress, at Heidelberg, 1888, and in 1889 E. Meyer, from the same clinic, continued the study of the subject. These two writers discussed more in detail the vulnerability of the macular region to various causes rather than to any particular traumatic cause. Their subsequent writings seem more of a clinical demonstration of their views previously set forth.

In 1894, Dr. E. C. Rivers published a very interesting case in *Knapp's Archives* on "Injury to the Eyes from Heavy Charge of Electricity." This case condensed is as follows: H. E., aged twenty-five years, employed as engineer in the motor house of the Denver Electric Car Company, accidentally made a short circuit with a steel wrench in his hand, causing the full 550 volts to pass through his arm and out at the wrench, accompanied by a loud report and a very intense flash. The patient was stunned and immediately had severe pain in his eyes.

Rivers saw the patient one hour after the accident and found the following condition present: Face with eyebrows and lids, neck, and hand badly burned. On examining the eyes the conjunctiva was found strongly congested and the cornea of both eyes almost opaque. Vision = perception of light. Under the use of atropine, castor-oil and dry aseptic cloths, the eyes progressed well.

On the third day the whole eschar on the cornea was thrown off and this being only the epithelial membrane the remainder was left clear. A few days later, L. E., V.$=^{20}/_{XL}$; R. E., V.$=^{20}/_{CC}$. The ophthalmoscope showed the media clear. The retina in the right eye looked hazy. Visual fields normal for all colors. Six months later the patient reported that he still suffered with photophobia and has to wear colored glasses. Is compelled to look very intently in order to see small objects. In my own cases the flash was not near enough to produce burning of the tissues.

Dr. L. D. Broose, of Evansville, Ind., has reported two cases of retinal irritation from electric flashes which correspond more in detail with my own:

J. H., aged twenty-eight years, an employee of an electric street railroad, accidentiy received a shock from one of the trolley wires and at the same time the eyes were subjected to a very intense flash of light. When seen five hours later the conjunctival vessels were found injected, the eyes suffused with tears, pupils contracted and patient suffering with intense pain. None of the parts were burned. Patient was directed to stay in a darkened room and a 2 per cent. solution of cocain was used in both eyes. In twenty-four hours he was able to resume work.

F. W., aged twenty-four years, also an employee of an electric street railroad, struck a live wire with a file which he held in his hand and immediately there was an intense flash and the patient rendered unconscious, this latter condition persisting but for a short time. When seen a few hours later the patient was complaining of severe pain in his eyes. The ball and lids were found markedly congested. Pupils contracted. Treated in the same manner as the first patient and in five days the patient was able to resume work. The ophthalmoscope showed nothing abnormal. No mention was made of the field of vision.

Professor Haab, in the July number of the *Monatsb. f. Augenheilkunde*, discusses anew this subject and reports another case whose clinical study presents some features of interest. As this article has not appeared in English I will take the liberty here of making an abstract of the report:

H. W., aged thirty-four years, machinist, consulted Professor Haab on August 9, 1895, concerning his eye, which had received a flash of light from a dynamo of about sixty ampères. He came the same morning immediately after the injury because of the dimness of vision noticed, especially in the lower field. He was stunned by the flash of light which was very near the eyes, and for a few moments he could see nothing. The right eye being nearer to the dynamo than the left, it was the most affected. The pain was severe in both, just as if needles were sticking them.

Examination: There was only a slight irritation with some hyperemia of the conjunctiva in right eye. Both pupils contracted, the right more than the left. The irides appeared normal. R. E., $V. = {}^{3}/_{XVIII}$; no improvement with glasses; L. E., V.= normal. The ophthalmoscope showed nothing abnormal. On the next day the condition was decidedly better. R. E., $V. = {}^{3}/_{X}$ w. — 0.5 $D. = {}^{3}/_{VI}$. On dilating the pupil the left eye showed nothing abnormal. In the right eye a very interesting condition was present:

1. Extending over the whole macular region there was a very fine milky discoloration so that the normal granular appearance was absent. In the deepest part of the macular center the discoloration was slightly less. A similar milky discoloration was nowhere else present in the retina.

2. Running along the periphery of the retina could be seen a few yellowish-white spots of irregular form and size about once or twice as broad as a large branch of the central artery. . . . Some spots existed also below the fovea, the latter being quite fine. In the middle of the fovea two very small bright specks were found. Whether these small spots were in the retina or in the pigment epithelium could not be determined. However, they were nowhere as white as those found in diabetes or albuminuria. Along the retinal vessels and on the papilla nothing abnormal could be seen. The lens and vitreous were normal. The treatment consisted in absolute rest and the wearing of dark glasses.

August 16. R. E., $V. = {}^{3}/_{VI}$ w. + I D; L. E., V.=normal. The macula is still slightly opaque.

October 13. The eye appeared normal. No sign of cataract.

These cases, as well as the subject itself, are of interest to the ophthalmologist as affording clinical data upon which to base a prognosis whenever such accidents should come under his own professional care. Since electricity has become so universal in domestic use, accidents referable to it as a cause will necessarily be more common. The prognosis in all diseased conditions is determined by clinical experience, and hence the greater the number of cases reported the more certainty there is for a basis of deduction. The writer of this article does not lay claim to any original investigation, but purposes to bring forth from others their own personal experience. In the study of the cases above reported there are many points of interest involved, and chief among them as to whether the changes produced in the eye had a chemical or a traumatic basis. This point certainly opens up an interesting field for investigation.

Another point to be noted was the universal presence of two symptoms in all the cases, viz.:

1. The contraction of the pupil from the retinal irritation which was so strong that this contraction persisted for days.

2. The pain, which always came on several hours after the accident.

